# Human MuStem cells repress T-cell proliferation and cytotoxicity through both paracrine and contact-dependent pathways

**DOI:** 10.1186/s13287-021-02681-3

**Published:** 2022-01-10

**Authors:** Marine Charrier, Judith Lorant, Rafael Contreras-Lopez, Gautier Téjédor, Christophe Blanquart, Blandine Lieubeau, Cindy Schleder, Isabelle Leroux, Sophie Deshayes, Jean-François Fonteneau, Candice Babarit, Antoine Hamel, Armelle Magot, Yann Péréon, Sabrina Viau, Bruno Delorme, Patricia Luz-Crawford, Guillaume Lamirault, Farida Djouad, Karl Rouger

**Affiliations:** 1 INRAE, Oniris, PAnTher, UMR 703, Oniris - Site de La Chantrerie, 101, Route de Gachet, CS. 40706, 44307 Nantes, France; 2 L’institut du Thorax, INSERM, CNRS, UNIV Nantes, 44007 Nantes, France; 3 Université de Nantes, Nantes, France; 4 INSERM U1183 IRMB, Hôpital Saint Eloi, CHRU Montpellier, Université de Montpellier, 80, Rue Augustin Fliche, 34295 Montpellier, France; 5 Laboratorio de Immunología Celular y Molecular, Facultad de Medicina, Universidad de los Andes, Las Condes, Chile; 6 CNRS, INSERM, CRCINA, Université de Nantes, 44000 Nantes, France; 7 INRAE, Oniris, IECM, 44307 Nantes, France; 8 Service de Chirurgie Infantile, Centre Hospitalier Universitaire (CHU) de Nantes, 44093 Nantes, France; 9 Laboratoire d’Explorations Fonctionnelles, Centre de Référence Maladies Neuromusculaires AOC, CHU Nantes, 44093 Nantes, France; 10 Biotherapy Division, Macopharma, 59420 Mouvaux, France; 11 IMPACT, Center of Interventional Medicine for Precision and Advanced Cellular Therapy, Santiago, Chile

**Keywords:** Muscular dystrophy, Cell therapy, Human adult stem cell, MuStem cell, Immunomodulation, T-cell

## Abstract

**Background:**

Muscular dystrophies (MDs) are inherited diseases in which a dysregulation of the immune response exacerbates disease severity and are characterized by infiltration of various immune cell types leading to muscle inflammation, fiber necrosis and fibrosis. Immunosuppressive properties have been attributed to mesenchymal stem cells (MSCs) that regulate the phenotype and function of different immune cells. However, such properties were poorly considered until now for adult stem cells with myogenic potential and advanced as possible therapeutic candidates for MDs. In the present study, we investigated the immunoregulatory potential of human MuStem (hMuStem) cells, for which we previously demonstrated that they can survive in injured muscle and robustly counteract adverse tissue remodeling.

**Methods:**

The impact of hMuStem cells or their secretome on the proliferative and phenotypic properties of T-cells was explored by co-culture experiments with either peripheral blood mononucleated cells or CD3-sorted T-cells. A comparative study was produced with the bone marrow (BM)-MSCs. The expression profile of immune cell-related markers on hMuStem cells was determined by flow cytometry while their secretory profile was examined by ELISA assays. Finally, the paracrine and cell contact-dependent effects of hMuStem cells on the T-cell-mediated cytotoxic response were analyzed through IFN-γ expression and lysis activity.

**Results:**

Here, we show that hMuStem cells have an immunosuppressive phenotype and can inhibit the proliferation and the cytotoxic response of T-cells as well as promote the generation of regulatory T-cells through direct contact and via soluble factors. These effects are associated, in part, with the production of mediators including heme-oxygenase-1, leukemia inhibitory factor and intracellular cell adhesion molecule-1, all of which are produced at significantly higher levels by hMuStem cells than BM-MSCs. While the production of prostaglandin E2 is involved in the suppression of T-cell proliferation by both hMuStem cells and BM-MSCs, the participation of inducible nitric oxide synthase activity appears to be specific to hMuStem cell-mediated one.

**Conclusions:**

Together, our findings demonstrate that hMuStem cells are potent immunoregulatory cells. Combined with their myogenic potential, the attribution of these properties reinforces the positioning of hMuStem cells as candidate therapeutic agents for the treatment of MDs.

**Supplementary Information:**

The online version contains supplementary material available at 10.1186/s13287-021-02681-3.

## Background

Muscular dystrophy (MD) is a heterogeneous group of more than 50 genetically distinct neuromuscular diseases characterized by local or generalized fiber degeneration [1]. While clinical presentation and severity can vary considerably, the progressive loss of muscle mass and function, leading to muscle weakness, reduced motility and premature death, is common to all forms [1]. As a consequence of molecular defects mainly affecting cytoskeletal or extracellular matrix proteins, muscle fiber membrane damage is common and results in profound disruption of homeostasis, as well as inflammation and fibrosis [2]. Duchenne muscular dystrophy (DMD) is the most common and devastating form of MD, affecting 1 in 3500–5500 male newborns [3, 4]. DMD is caused by mutations in the gene encoding dystrophin, leading to a lack of a functional protein essential for maintenance of muscle fiber integrity [5].

The presence of muscle inflammation and a significant increase in the proportion of immune cells in dystrophic muscle reveals an essential functional role of the immune system in the pathogenesis of MD [6, 7]. This association was initially supported by studies demonstrating that glucocorticosteroid-mediated immunosuppression limits clinical signs and delays the course of DMD in patients and animal models [8, 9]. The diversity of leukocytes that infiltrate dystrophic muscle, including neutrophils, eosinophils, macrophages, helper CD4^+^ T-lymphocytes and cytotoxic CD8^+^ T-lymphocytes (CTLs), further supports an immunological component [10–12]. In X-chromosome-linked muscular dystrophy (*mdx*) mice, one of the most commonly used animal models of DMD [13], depletion of myeloid cells at an early age markedly decreases the proportion of necrotic fibers [11, 12]. T-cells are among the first cells to infiltrate *mdx* mouse muscle, prior to the onset of necrosis, indicating an important role as effectors in early disease pathogenesis [14]. In young *mdx* mice, selective T-cell inhibition markedly diminishes the extent of inflammatory cell infiltrate and reduces both muscle necrosis and fibrosis, underscoring the importance of the adaptive immune system in DMD. Furthermore, disease severity is reduced in *mdx* mice after pharmacological inhibition or genetic ablation of the inflammatory cytokines interferon-γ (IFN-γ) and tumor-necrosis factor-α (TNF-α) [15, 16]. Finally, accumulation of regulatory T cells (Tregs) has been demonstrated in infiltrates in muscle tissue from DMD patients [17], *mdx* mice [17, 18] and dysferlin-deficient mice [19]. These findings highlight the therapeutic importance of regulating the infiltration and accumulation of immune cells in dystrophic muscle, both of which contribute to disease progression and severity.

Over the last two decades, muscle repair potential has been attributed to tissue-resident stem cells capable of forming new muscle fibers in response to acute injury. These cells include side population (SP) cells [20, 21], CD133^+^ cells [22, 23], mesoangioblasts (Mabs) [24–26], bone-marrow- and adipose tissue-derived-mesenchymal stem cells (BM-MSCs and AD-MSCs, respectively) [27, 28], PW1^+^/Pax7^+^ interstitial cells (PICs) [29] and muscle-derived stem cells (MDSCs) [30, 31]. The newly discovered potential of these adult stem cells has opened novel therapeutic avenues for MDs in addition to strategies targeting dystrophin restoration including exon skipping with antisense oligonucleotides, vector-mediated gene therapy and CRISPR/Cas9-mediated gene editing [32]. In particular, it constitutes a promising means of overcoming the limited efficacy of myoblast transplantation. Moreover, most if not all MSCs are immunoprivileged. MSCs lack human leukocyte antigen type-II (HLA-II) and co-stimulatory molecules CD80 and CD86, which are required for T-lymphocyte activation, but express HLA-I on their surface [33]. An ability to escape immune recognition has been described for human AD-MSCs in immunocompetent animal models of DMD and allows long-term integration of these cells into muscle tissue [28, 34]. MSCs are also potent immunoregulatory cells and appear to be capable of regulating both innate and adaptive immune responses in vitro and in vivo [35–38]. Moreover, MSCs control the phenotype and immunological responses of T-cell subsets (helper CD4^+^, CTLs, Treg cells) [38–42], B-cells [43], natural killer (NK) cells [44, 45], dendritic cells (DC) [46, 47] and macrophages [48, 49]. In vitro, MSCs suppress T-cell proliferation in response to mitogens, alloantigens and activating antibodies [39, 50] and promote the generation of Tregs [38, 51]. In vivo, MSCs enhance long-term allograft acceptance and tolerance [40, 52] and exert therapeutic effects in experimental models of inflammatory and autoimmune disorders [53, 54]. These immunomodulatory/immune-dampening properties of MSCs involve direct contact with target cells [55, 56], but are largely mediated by the release or synthesis of numerous factors [57], the best described of which are indoleamine 2,3-dioxygenase (IDO) [58], prostaglandin E2 (PGE2) [59, 60], inducible nitric oxide synthase (iNOS) [61] and heme oxygenase-1 (HO-1) [62].

In the past few years, we have characterized a type of MDSC isolated from tissue samples from healthy dogs and humans. Based on their phenotype and plasticity, we have shown that these cells, referred as MuStem cells, correspond to early myogenic-committed progenitors with a mesenchymal perivascular profile. Importantly, these oligopotent stem cells display an interesting potential for skeletal and cardiac muscle repair [63–66]. After allogeneic transplantation into golden retriever muscular dystrophy (GRMD) dogs, a clinically relevant animal model of DMD, MuStem cells contribute to muscle fiber formation, induce long-term muscle fiber regeneration and limit the progression of muscle damage and fibrosis [63, 67]. Similarly, human MuStem (hMuStem) cells show in vivo a robust capacity for muscle regeneration after delivery into injured skeletal muscle in immunodeficient mice [64] and efficiently counteract adverse tissue remodeling, primarily by limiting fibrosis, in an immunodeficient rat model of myocardial infarction [66]. While these findings have positioned hMuStem cells as an attractive tool for muscle regenerative medicine, their immunoregulatory properties, which are pivotal for tissue remodeling, have not been investigated to date.

In the present study, we investigated whether hMuStem cells can exert immunosuppressive effects by regulating the immune response. We characterized the immunophenotype of these cells and compared it with that of BM-MSCs. Moreover, we studied the effect of hMuStem cells on T-cell proliferation and cytotoxicity in co-culture experiments. These original data provide new insights into the modes of action of hMuStem cells and reinforce their potential as an effective therapeutic agent for the treatment of MDs.

## Methods

### Human skeletal muscle tissue

Tissue samples were obtained from *Paravertebralis* muscle biopsies collected from patients aged 12–19 years. Patients were free of known muscle disease and had undergone surgery for acute scoliosis at the Department of Pediatric surgery of the Centre Hospitalier Universitaire (CHU) de Nantes (France). Written informed consent was obtained from all patients. All protocols were approved by the Clinical Research Department of the CHU (Nantes, France), according to the rules of the French Regulatory Health Authorities (Approval Number: MESR/DC-2010-1199). The biological sample bank was created in compliance with national guidelines regarding the use of human tissue for research (Approval Number: CPP/29/10).

### Isolation and culture of human MuStem cells

Human MuStem cells were independently isolated from muscle biopsies from 5 patients and cultured, as previously described [64, 66]. For pro-inflammatory stimulation, cells were expanded until they reached approximately 60–70% confluence, and then the medium was changed for growth medium containing 50 ng/mL of both TNF-α and IFN-γ (Miltenyi, Bergisch Gladbach, Germany) and cultured for 24 h.

### Isolation and culture of human bone marrow-derived mesenchymal stem cells

Human BM-MSCs were collected from 4 patients aged 5, 11, 19 and 60 years who underwent hip replacement surgery. The patients were informed and provided written informed consent prior to collection of tissue samples as approved by the French Ministry of Higher Education and Research (DC-2010-1185). BM-MSCs were cultured, characterized at the phenotypic level and tested for their tri-lineage differentiation potential as previously described [68, 69]. For pro-inflammatory stimulation, cells were expanded until they reached approximately 60–70% confluence, and then the medium was changed for fresh medium containing 50 ng/mL of both TNF-α and IFN-γ (Miltenyi) and cultured for 24 h.

### Immunosuppression assay

Three sets of independent experiments were conducted with hMuStem cells that were successively co-cultured with allogeneic human PBMCs, human CD3^+^ lymphocytes/allogeneic PBMCs and human CD8^+^ T-cell clones. A detailed description of these immunosuppression assays is provided in Additional file 5: Methods.

### Cytotoxicity assay

Meso 34 NanoLuc cells were seeded at 5 × 10^3^ cells/well in 96-well plates [70, 71]. After 3 h, 1 × 10^4^ clonally-derived CD8^+^ T-cells previously co-cultured with either hMuStem cells, BM-MSCs or their respective secretome were added. To determine their cytotoxic effects on Meso 34 NanoLuc cells, 45 μL of medium was collected after 24 h and light emission was measured at 480 nm immediately after addition of 5 μL of 30 μM coelenterazine using a Mithras LB 940 microplate analyzer (Berthold Technologies, Baden Württemberg, Germany), which measures nanoluciferase activity released in the supernatant following cell lysis.

### Flow cytometry

Human MuStem cells, BM-MSCs, PBMCs or MUC-1-specific CD8^+^ T-cells were resuspended in cold phosphate buffered saline (PBS)/2% human serum and 1 × 10^5^ cells were incubated (30 min, 4 °C) in darkness with fluorochrome-conjugated Ab at a saturating concentration. The Abs used are listed in Additional file 4: Table S1. Isotype-matched Ab and fluorescence minus one-control samples were used as negative controls for gating and analyses. Where applicable, 7-amino-actinomycin D (7-AAD; BD Biosciences, Franklin Lakes, NJ, USA) was added to evaluate cell viability. For immunophenotyping of hMuStem cells, samples were acquired using a FACS Aria flow cytometer (BD Biosciences), and for analyses of PBMCs and CD8^+^ T-cells a FACS Canto II (BD FACSDivaTM Software) was used. For each experiment and labeling, at least 15 × 10^3^ viable cells were considered. All the collected data were analyzed using FlowJo software (FlowJo, Ashland, OR, USA).

### Immunocytochemistry

Human macrophages, obtained from the Clinical Transfer Facility of the CHU de Nantes (CICBT0503, Nantes, France), were seeded at the density of 1.5 × 10^5^ cells/cm^2^ in 12-well plates and cultured in RPMI 1640 (Thermo Fischer Scientific, Illkirch, France) containing 10% human serum (EFS, Nantes, France), 1% glutamin (Sigma-Aldrich, Saint Quentin-Fallavier, France), 1000 IU/mL M-CSF (Miltenyi) and 1% 10,000 IU/mL penicillin, 10 mg/mL streptomycin, 25 µg/mL fungizone (amphotericin B) (PSF; Sigma-Aldrich). After 72 h, they were activated with 100 ng/mL of lipopolysaccharide (LPS) from *Escherichia coli* 0111:B4 (Sigma-Aldrich) for 24 h. RAW 264.7 murine macrophage cell line (ATCC TIB-71, Manassas, VA, USA) was provided by the cell bank of the IECM Lab (Nantes, France). It was seeded at the density of 2.2 × 10^4^ cells/cm^2^ in 12-well plates and cultured in RPMI 1640 (Gibco) containing 10% fetal calf serum (FCS), 1% glutamin (Sigma-Aldrich), 1% PSF (Sigma-Aldrich) during 96 h. Human MuStem cells, BM-MSCs, human macrophages or RAW cells were fixed in cold methanol (15 min, − 20 °C) and treated with 0.3% triton X-100 (30 min, 4 °C). After incubation (1 h, RT) in blocking buffer (5% goat serum in PBS), cells were incubated overnight with iNOS Ab (1:100, sc-651 clone, Santa Cruz Biotechnology, Santa Cruz, CA, USA) and counterstained (15 min, 37 °C) with DAPI fluorescent cell-permeable DNA probe (Life Technologies Ltd, Paisley, UK). The number of positive cells was determined using Fiji image analysis software [72]. For each condition, more than 300 cells were counted.

### Reverse transcription and real-time semi-quantitative PCR

Total RNA was extracted using the RNeasy mini or micro kit following the manufacturer’s instructions (Qiagen, Santa Clara, CA, USA), quantified using a NanoDrop spectrophotometer (Labtech, Wilmington, DE, USA) after DNase treatment (Ambion, Austin, TX, USA) and converted to cDNA by reverse transcription as described previously [64]. Oligonucleotide primers used for semiquantitative RT-PCR analysis of gene expression were designed using Oligo Primer Analysis Software v.7 (Molecular Biology Insights Inc., Colorado Springs, CO, USA) and are listed in Additional file 4: Table S2. Data were normalized to mRNA levels of the housekeeping gene RPS18 and were calculated using the 2–∆Ct method.

### ELISA assay

Supernatant of CTLs, hMuStem cells, or BM-MSCs (cultured under either basal or TNF-α/IFN-γ-stimulated conditions) was collected, centrifuged to remove any cell fragments and stored at − 20 °C. The presence of secreted proteins, specifically granzyme B, hepatocyte growth factor (HGF), VEGF, PGE2, IL-6, IL-8, IL-10, LIF and Gal-1 was measured by ELISA (granzyme B, VEGF, IL-6, IL-8, IL-10, LIF, Gal-1: DuoSet ELISA R&D Systems; PGE2: Enzo Life Sciences; IL35: Wuhan Fine Biological Technology, Tebu bio-SAS, Le Perray En Yvelynes, France), according to the manufacturer’s instructions.

### Statistical analysis

All data are reported as the mean ± SEM. Lymphocyte proliferation was compared between co-cultures containing different proportions of MuStem cells using a Kruskal–Wallis test followed by Dunn’s multiple comparisons between pairs. Expression and secretion of regulatory molecules by hMuStem cells was compared with that of BM-MSCs using the Mann–Whitney U test. Expression and secretion of regulatory molecules by hMuStem cells expanded under stimulated or unstimulated conditions was compared using the Wilcoxon matched-pairs signed rank test. A value of *p* < 0.05 was considered statistically significant.

## Results

### hMuStem cells display a poorly immunogenic and immunosuppressive phenotype

**Fig. 1:**
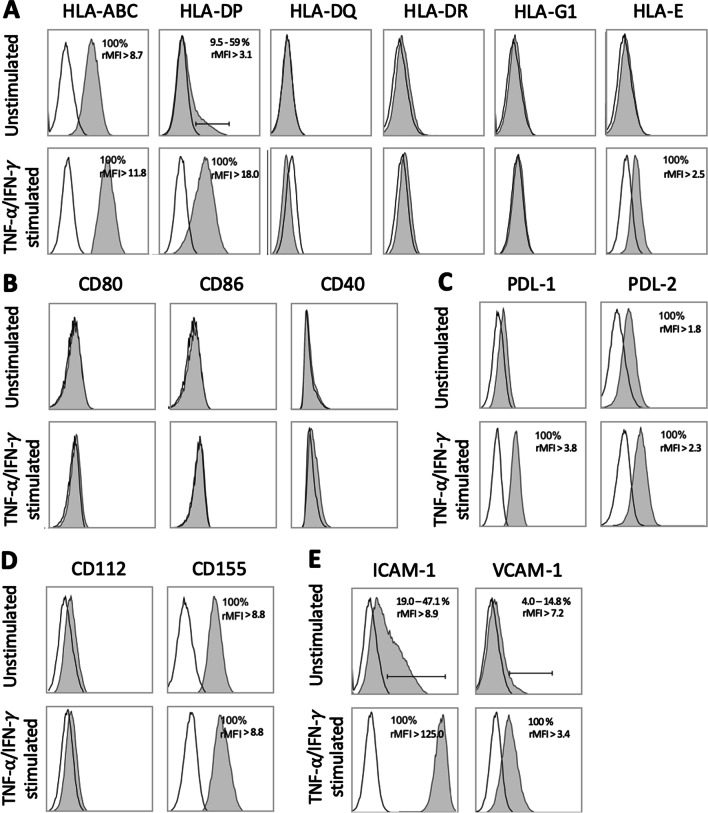
Phenotypic profile of human MuStem cells focused on immune-cell-related markers. Cell events were first selected upon their size and granularity (forward scatter vs. side scatter density plot), while the FSC-A/FSC-H plot on gated events allowed identification of single cells. Flow cytometry comparison of: **a** HLA class I (HLA-ABC, HLA-G1 and HLA-E) and class II (HLA-DQ, -DR and -DP) molecules; **b** co-stimulatory molecules CD80, CD86 and CD40; **c** programmed death ligand (PDL)-1 and -2; **d** TIGIT receptor CD112 and CD155; and **e** intracellular cell adhesion molecule-1 (ICAM-1) and vascular cell adhesion molecule (VCAM-1) in hMuStem cells cultured in basal condition (unstimulated) or after stimulation with TNF-α/IFN-γ (pro-inflammatory condition). When an expression is detected, mean ± SEM of positive cells and rMFI are reported on the top right corner. Results of one representative cell batch out of five independent batches are presented

To determine whether the hMuStem cells display an immunosuppressive phenotype similar to BM-MSCs [33, 73, 74], we assessed the expression of HLA-I and -II, as well as molecules involved in T-cell interaction. Human MuStem cells from 5 independent donors were expanded in vitro and analyzed at passage 5 (P5) using fluorescence-activated cell sorting (FACS). All hMuStem cells shared a typical expression pattern of myogenic progenitors with a signature of perivascular MSCs (Additional file 2: Figure S1), in agreement with our previous findings [64, 66, 75]. Given that stimulation with pro-inflammatory cytokines modifies the phenotype of MSCs [73], hMuStem cells were cultured either in basal media or in presence of TNF-α and IFN-γ. While hMuStem cells constitutively expressed HLA-I molecules (HLA-ABC, 100%) and HLA-DP (29.2% ± 23.9%; range 9.5–59.0%), they were uniformly negative for HLA-DQ and HLA-DR (Fig. 1A; Table 1). Moreover, they did not express HLA-G1 or HLA-E, which are involved in immune tolerance and NK cell inhibition, respectively. Human MuStem cell stimulation with TNF-α/IFN-γ increased HLA-ABC expression (relative mean fluorescence intensity [rMFI]: 21.2 ± 10.3 vs. 13.3 ± 7.3 in basal conditions) and resulted in HLA-DP expression in 88.3% ± 33.2% of cells. Stimulation with TNF-α/IFN-γ had no effect on expression levels of HLA-DQ, HLA-DR or HLA-G1. Notably, HLA-E expression was induced in all hMuStem cells in response to stimulation with TNF-α/IFN-γ.

**Table 1:** Expression levels of immune cell-related markers measured in human MuStem cells

Cell surface markers	Unstimulated hMuStem cells		TNF-α/IFN-γ-stimulated hMuStem cells	
	Positive cells (%)	rMFI	Positive cells (%)	rMFI
*Human leukocyte antigen*				
HLA-ABC	100%	13.3 ± 7.3	100%	21.2 ± 10.3
HLA-DP	29.2% ± 23.9%	9.7 ± 4.8	88.3% ± 23.5%	19.6 ± 10.5
HLA-E	0%	NA	100%	4.0 ± 1.1
*Immune-checkpoint ligands*				
Co-inhibitory factors				
PDL-1	0%	NA	100%	4.0 ± 1.3
PDL-2	100%	2.3 ± 0.3	100%	3.7 ± 1.1
*Other factors*				
CD155	100%	12.0 ± 2.9	100%	14.0 ± 3.6
*Adhesion molecules involved in T-cell interaction*				
ICAM-1	37.4% ± 11.2%	10.4 ± 6.0	100%	284.5 ± 124.8
VCAM-1	8.3% ± 4.0%	6.0 ± 3.7	100%	5.6 ± 3.2

Flow cytometry comparison of human leukocyte antigen (HLA) molecules, immune-checkpoint ligands and adhesion molecules involved in T-cell interaction in hMuStem cells cultured in basal (unstimulated) or pro-inflammatory (TNF-α/IFN-γ-stimulation) conditions. Data are presented as the mean ± SEM (*n* = 5, independent batches) percentage of positive events for each marker and the relative mean fluorescence intensity (rMFI). NA, not applicable

We next examined the expression pattern of molecules involved in the regulation of T-lymphocyte activity. All hMuStem cells were homogeneously negative for the co-stimulatory molecules CD80, CD86 and CD40, which are required for efficient T-cell activation, even after stimulation with TNF-α/IFN-γ (Fig. 1B). Human MuStem cells did not express the inhibitory lymphocyte molecule corresponding to programmed death ligand-1 (PDL-1), but homogeneous low-level PDL-2 expression was detected under basal conditions (Fig. 1C). Interestingly, stimulation with TNF-α/IFN-γ induced uniform PDL-1 expression by hMuStem cells and significantly enhanced the intensity of PDL-2 expression (rMFI: 3.7 ± 1.1 vs. 2.3 ± 0.3 in basal conditions), as previously described for BM-MSCs [76]. We next investigated the expression of CD112 and CD155, two molecules expressed by antigen-presenting cells and known to mediate inhibition of lymphocytes by their interaction with TIGIT [77]. While CD112 expression was not detected in either basal or stimulated conditions, hMuStem cells showed constitutive CD155 expression, which was unaffected by stimulation with TNF-α/IFN-γ **(**Fig. 1D). Finally, in basal conditions hMuStem cells expressed two molecules known to induce lymphocyte adhesion: intracellular cell adhesion molecule-1 (ICAM-1) and vascular cell adhesion molecule-1 (VCAM-1) (37.4% ± 11.2% and 8.3% ± 4.0%, respectively) (Fig. 1E). Stimulation with TNF-α/IFN-γ significantly increased the expression of both ICAM-1 and VCAM-1 (100% positivity for both molecules), and the first in a much higher extent as appreciated by a strongly shifted rMFI (Fig. 1E).

Together, these data suggest that hMuStem cells are poorly immunogenic cells considering the expression of HLA, co-stimulatory and inhibitory molecules and that they interact with T-cells such as BM-MSCs.

### hMuStem cells inhibit T-lymphocyte proliferation and induce Treg-like cells in peripheral blood mononuclear cells

**Fig. 2:**
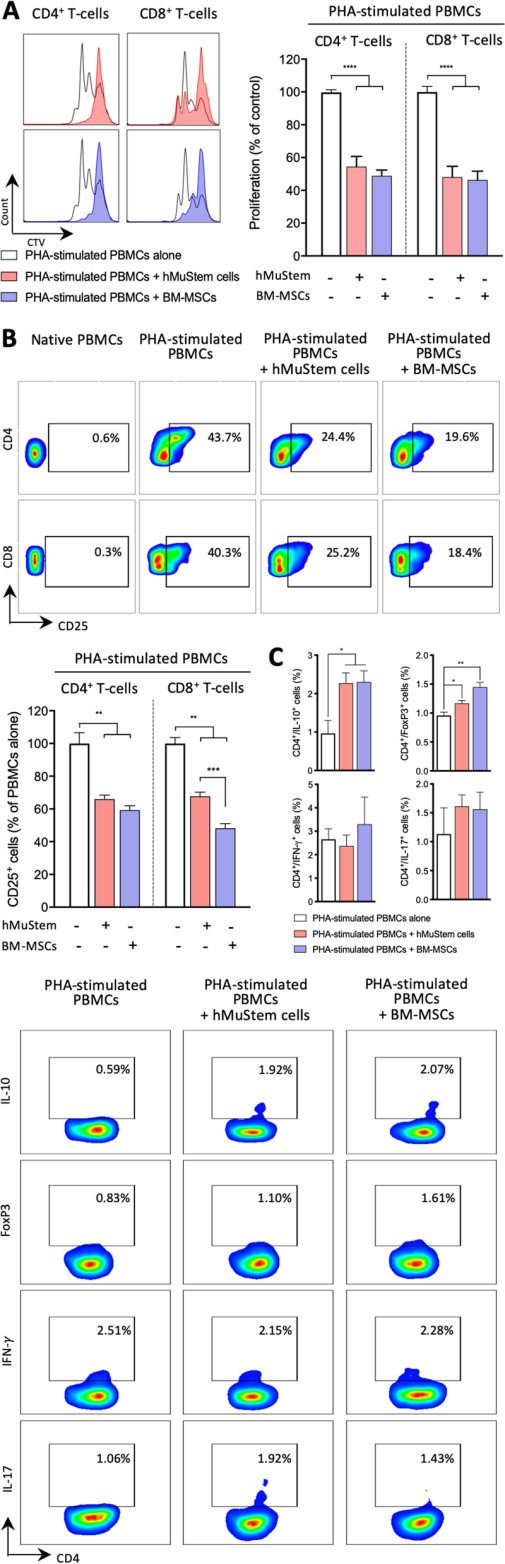
Effect of human MuStem cells and bone marrow-derived mesenchymal stem cells on proliferation and phenotype of T-cells in peripheral blood mononucleated cells. **a** Representative profile (left panel) and quantification (right panel) of the proliferation of Cell Trace Violet (CTV)-labeled CD4^+^ and CD8^+^ T-cells in peripheral blood mononucleated cells (PBMCs) cultured alone or with unstimulated hMuStem cells (*n* = 5, independent batches) or bone marrow-derived mesenchymal stem cells (BM-MSCs; *n* = 4, independent batches). PBMCs were stimulated with phyto-hemagglutinin (PHA) to induce T-cell proliferation. The suppressive capacity of hMuStem cells or BM-MSCs was determined by tracking cell division of CTV-labeled PBMCs. **b** Representative FACS plots with gating of CD25^+^ cells in CD4^+^ and CD8^+^ T-cell fractions of PBMCs cultured alone or under PHA stimulation and with hMuStem cells or BM-MSCs. Percentages are expressed as the percentage of PBMCs cultured alone. **c** Representative FACS plots with gating of IL-10^+^, FoxP3^+^, IFN-γ^+^ and IL-17^+^ cells in CD4^+^ T-cell fractions of PBMCs cultured under PHA stimulation only and with hMuStem cells or BM-MSCs. Percentages are expressed as the percentage of PBMCs cultured alone. Data are presented as mean ± SEM (*p < 0.05, **p < 0.01, **p < 0.001, ****p < 0.0001; Mann–Whitney U test)

Based on the phenotypic features of hMuStem cells, we sought to determine whether they could exert immunosuppressive effects on T cells. PHA-stimulated allogeneic peripheral blood mononuclear cells (PBMCs) previously stained with cell trace violet (CTV) were co-cultured for 3 days with hMuStem cells (*n* = 5, independent donors) or BM-MSCs, used as reference (*n* = 4, independent donors), at a ratio of 1:10 PBMCs. Compared with PBMC-only cultures, hMuStem cells resulted in significant inhibition of both CD4^+^ and CD8^+^ T-cell proliferation, as evidenced by proportions of proliferating cells of 54.5% ± 28.0% and 48.1% ± 29.9%, respectively (*p* < 0.001; Fig. 2A). Similar results were obtained for BM-MSCs with 48.9% ± 13.0% and 46.3% ± 21.1% of CD4^+^ and CD8^+^ T cells, respectively.

Expression of the T-cell activation marker CD25 decreased when hMuStem cells were co-cultured with PBMCs (66.0% ± 7.2% and 67.7% ± 7.6% for CD4^+^ and CD8^+^ T-cells, *p* < 0.01; Fig. 2B). While a similar proportion of CD4^+^/CD25^+^ cells (59.2% ± 8.2% with respect to PBMC-only culture) was observed in BM-MSC + PBMC co-cultures, those of CD8^+^/CD25^+^ cells was lower (48.2% ± 8.5%), suggesting a stronger effect of BM-MSCs than hMuStem cells on CD8^+^ T-cell activation (*p* < 0.001).

To further investigate the effects of hMuStem cells on T-cells, we characterized the expression profile of the T-cell subset regulatory factors IL-10 and FoxP3 and the pro-inflammatory factors IFN-γ and IL-17 in CD4^+^ T-cells collected from hMuStem cell + PBMC and BM-MSC + PBMC co-cultures. Remarkably, the proportion of CD4^+^/IL-10^+^ cells and CD4^+^/FoxP3^+^ cells in hMuStem cell + PBMC co-cultures was significantly higher than that observed in PBMC-only cultures (*p* < 0.05; Fig. 2C). However, compared with hMuStem cells, BM-MSCs were more efficient at generating CD4^+^/FoxP3^+^ cells. Moreover, FACS analysis revealed that in hMuStem cell + PBMC and BM-MSC + PBMC co-cultures, the proportions of CD4^+^/IFN-γ^+^ cells and CD4^+^/IL-17^+^ T-cells were similar to those seen in PBMC-only cultures, indicating that hMuStem cells, like BM-MSCs, did not significantly affect the proportions of pro-inflammatory lymphocytes. Overall, these results demonstrate that hMuStem cells can significantly reduce proliferation and activation of CD4^+^ and CD8^+^ T-cells and exert an immunosuppressive effect by promoting a Treg cell-like phenotype in CD4^+^ T-lymphocytes without affecting their pro-inflammatory capacity.

### hMuStem cells express classical MSC immunomodulatory mediators

**Fig. 3:**
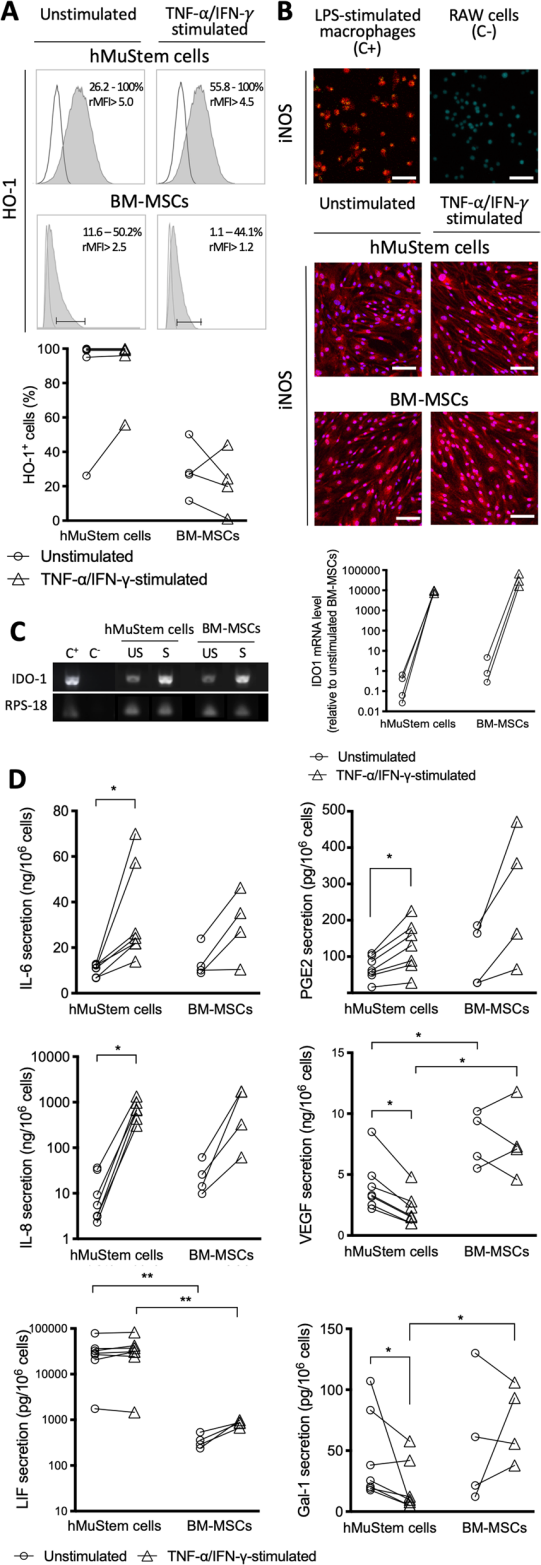
Expression and secretion of immunoregulatory mediators in cultured human MuStem cells and bone marrow-derived mesenchymal stem cells. **a** Flow cytometry comparison of heme oxygenase-1 (HO-1) expression in hMuStem cells and BM-MSCs. **b** Fluorescent immunolabeling of inducible nitric oxide synthase (iNOS) in hMuStem cells and BM-MSCs. Lipopolysaccharide (LPS)-human monocyte-derived activated macrophages for 24 h and RAW 264.7 cell line were used as positive (C+) and negative (C−) controls, respectively. Nuclei were counterstained with DAPI (blue). Scale bars, 100 µm. **c** Representative RT-PCR profile of indoleamine 2,3-dioxygenase-1 (IDO-1) gene obtained for hMuStem cells and BM-MSCs. For each sample, the level of IDO-1 expression was quantified using the average mRNA level of IDO-1 obtained in unstimulated BM-MSCs as a reference. LPS-human monocyte-derived activated macrophages for 24 h and water were used as positive (C+) and negative (C−) controls, respectively. **d** Interleukin, growth factor, enzyme and carbohydrate-binding protein secretion profile of hMuStem cells and BM-MSCs. ELISA assays were performed using culture supernatant collected 24 h after medium change. Results are expressed as individual values and normalized as concentration relative to 1 million cultured cells (ng or pg/10^6^ cells). Each experiment was performed on at least 5 and 4 independent batches of hMuStem cells and BM-MSCs, respectively. Stimulation corresponds to a 24-h treatment with 50 ng/mL of TNF-α and IFN-γ. *p < 0.05, **p < 0.01, **p < 0.001, ****p < 0.0001; Wilcoxon matched-pairs signed rank test (unstimulated vs. stimulated) or Mann–Whitney U test (hMuStem cells vs. BM-MSCs). US, unstimulated; S, TNF-α/IFN-γ-stimulated

To investigate the mechanisms underlying the suppressive activity of hMuStem cells, we examined the expression of a panel of well-described regulatory mediators including metabolic enzymes, pleiotropic hormones and interleukins using flow cytometry, immunocytochemistry and enzyme-linked immunosorbent assay (ELISA). In 4 out of 5 hMuStem cell batches, more than 95% of cells were positive for the heme oxygenase 1 (HO-1), whereas 26.2% HO-1^+^ cells were detected in the remaining batch (Fig. 3A). Remarkably, in BM-MSC batches the proportion of HO-1^+^ cells ranged from 11.6 to 50.2% (i.e., markedly lower than detected in hMuStem cells). Moreover, stimulation with TNF-α/IFN-γ induced a more than twofold increase in the proportion of HO-1^+^ cells in the hMuStem cell batch exhibiting initially non-homogenous expression for the metabolic enzyme, whereas no clear change was observed in stimulated BM-MSCs. Immunocytochemistry analysis revealed that hMuStem cells and BM-MSCs were both uniformly positive for the expression of iNOS (Fig. 3B). IDO-1 gene expression was detected in both hMuStem cells and BM-MSCs, and increased significantly (by a factor of 141,000 and 36,000 in hMuStem cells and BM-MSCs, respectively) upon stimulation with TNF-α/IFN-γ (Fig. 3C).

Human MuStem cells and BM-MSCs showed similar levels of IL-6 expression (10.4 ± 2.5 and 13.7 ± 6.8 ng/10^6^ cells, respectively). Stimulation with TNF-α/IFN-γ resulted in a significant increase of IL-6 secretion in some hMuStem cell batches (*p* < 0.05, Fig. 3D; Table 2). This increase was greater than that observed in BM-MSCs. IL-8 production was observed in all hMuStem cells (13.2 ± 14.8 ng/10^6^ cells) and increased significantly in response to stimulation with TNF-α/IFN-γ (640.2 ± 426.3 ng/10^6^ cells; *p* < 0.05). A similar profile was observed in BM-MSCs, with, however, a less pronounced impact of the stimulation. Neither IL-10 nor IL-35 were detected in hMuStem cell or BM-MSC culture supernatant. Moreover, in hMuStem cell batches secretion of leukemia inhibitory factor (LIF) was 90-fold higher than in BM-MSCs (*p* < 0.01). LIF secretion was unchanged by stimulation with TNF-α/IFN-γ in both hMuStem cells and BM-MSCs (Fig. 3C). Levels of PGE2 secretion were similar in hMuStem cells and BM-MSCs (6.9 ± 3.3 and 10.1 ± 8.5 pg/10^6^ cells, respectively), although greater variability was observed in BM-MSC batches. Following stimulation with TNF-α/IFN-γ, increased PGE2 secretion was observed for all batches of both cell types. In MuStem cells, stimulation resulted in a twofold increase (*p* < 0.05). Analysis of vascular endothelial growth factor (VEGF) secretion revealed significantly lower levels in hMuStem cells than BM-MSCs, both in basal conditions and following stimulation with TNF-α/IFN-γ (*p* < 0.05). Interestingly, stimulation induced a twofold decrease in VEGF secretion in hMuStem cells, whereas in BM-MSCs a variable response was observed. Finally, variable levels of Galectin-1 (Gal-1) secretion were detected in the different batches of hMuStem cells and BM-MSCs. In hMuStem cells, stimulation with TNF-α/IFN-γ resulted in a twofold decrease in Gal-1 levels compared with basal conditions (*p* < 0.05). Levels of secreted Gal-1 were lower in stimulated hMuStem cells than stimulated BM-MSCs (*p* < 0.05). Overall, our findings indicate that hMuStem cells, like BM-MSCs, produce inflammation-modulated soluble factors that exert immunomodulatory effects, including PGE2, IL-6, LIF, VEGF and Gal-1. The secretion profile of hMuStem cells was clearly impacted by pro-inflammatory conditions, as evidenced by concomitant increases in the secretion of PGE2, IL-6 and LIF and decreases in levels of VEGF and Gal-1. Human MuStem cells are distinguished from BM-MSCs by higher levels of HO-1 expression and LIF secretion and lower levels of VEGF secretion in basal conditions, and by decreased secretion of VEGF and Gal-1 in pro-inflammatory conditions.

**Table 2:** Secretory profile of immunoregulatory mediator by human MuStem cells and bone marrow-derived mesenchymal stem cells cultured in basal or pro-inflammatory conditions

	hMuStem cells		BM-MSCs	
	Unstimulated	TNF-⍺/IFN-γ-stimulated	Unstimulated	TNF-⍺/IFN-γ-stimulated
*Interleukins*				
IL-6 (ng/10^6^ cells)	10.4 ± 2.5	34.2 ± 22.0	13.7 ± 6.8	29.7 ± 15.0
IL-8 (ng/10^6^ cells)	13.2 ± 14.9	640.2 ± 426.3	27.9 ± 23.5	1 094.2 ± 730.1
LIF (pg/10^6^ cells)	32 200 ± 23 300	49 870 ± 29 500	355.4 ± 130.0	847.2 ± 126.7
*Growth factors*				
PGE2 (ng/10^6^ cells)	6.9 ± 3.3	12.7 ± 6.7	10.1 ± 8.5	26.4 ± 18.3
VEGF (ng/10^6^ cells)	4.1 ± 2.1	2.1 ± 1.3	7.8 ± 2.2	7.7 ± 3.0
*Other factors*				
Galectin-1 (pg/10^6^ cells)	44.4 ± 36	20.0 ± 21.1	56.2 ± 53.6	73.2 ± 31.8

The secretory profile was determined by ELISA of culture supernatant collected 24 h after medium change. Results are expressed as the mean ± SEM or range of the concentration relative to 10^6^ cultured cells. Each experiment was performed on 5 and 4 independent batches of hMuStem cells and bone marrow-derived mesenchymal stem cells (BM-MSCs), respectively. Stimulation corresponds to a 24-h treatment with 50 ng/mL TNF-α/IFN-γ

### hMuStem cells suppress T-cell proliferation through PGE2 secretion and iNOS activity

**Fig. 4:**
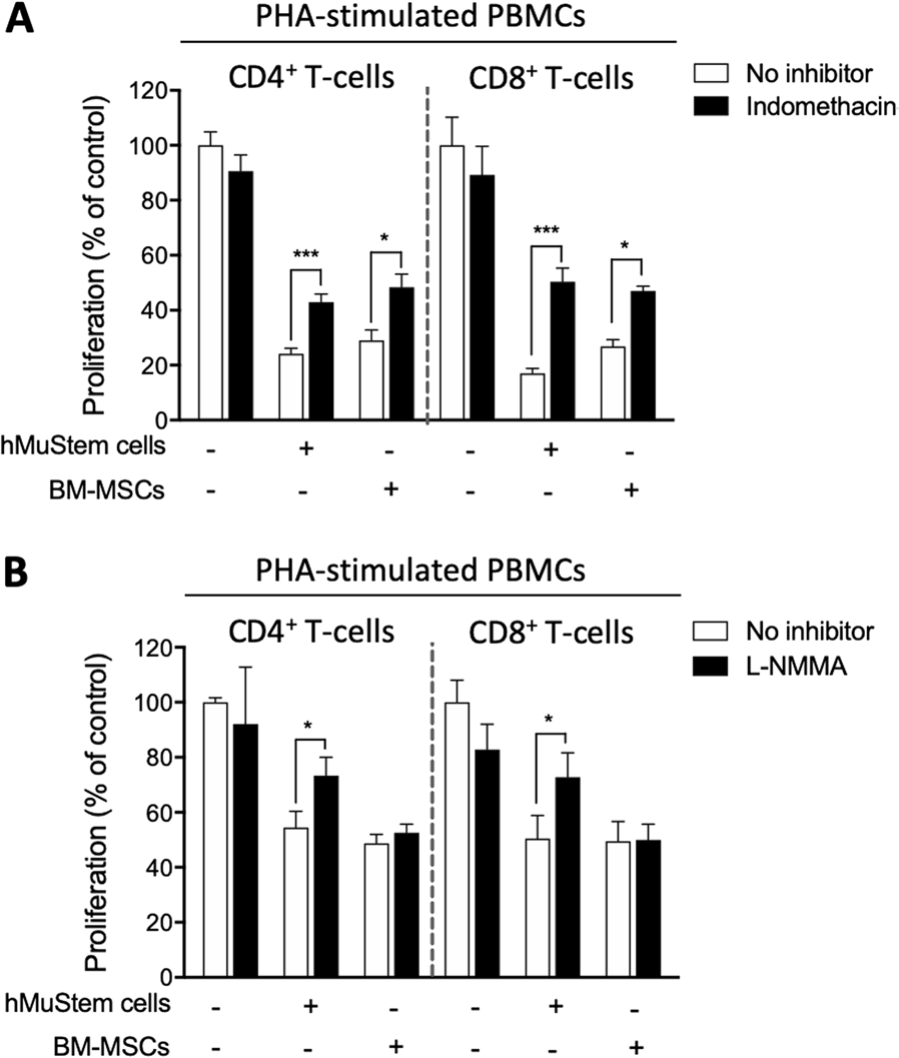
Involvement of prostaglandin E2 secretion and inducible nitric oxide synthase activity in human MuStem cell-mediated T-cell inhibition. Quantification of the proliferation of Cell Trace Violet (CTV)-labeled CD4^+^ and CD8^+^ T cells in co-cultures of naïve hMuStem cells + PBMCs or naïve BM-MSCs + PBMCs **a** with or without indomethacin, a prostaglandin E2 (PGE2) inhibitor; and **b** with or without NG-monomethyl-L-arginine (L-NMMA), an inducible nitric oxide synthase (iNOS) inhibitor. Experiments were performed on 5 and 4 independent batches of hMuStem cells and BM-MSCs, respectively. Data are presented as the mean ± SEM (*p < 0.05, ***p < 0.001; Mann–Whitney U test)

To clarify the mechanism underlying the immunosuppressive effects of hMuStem cells on lymphocyte proliferation, we performed a series of inhibitory assays. Indomethacin, a selective inhibitor of cyclooxygenase-2, which mediates PGE2 synthesis, was added to co-cultures of hMuStem cells + PHA-stimulated PBMCs, after first verifying that indomethacin does not interfere with PBMC proliferation in PBMC-only cultures. BM-MSCs were also considered in the experiment. In indomethacin-treated co-cultures of hMuStem cells + stimulated PBMCs, the proportions of proliferative CD4^+^ and CD8^+^ T-lymphocytes were significantly higher than those observed in untreated co-cultures (42.9% ± 8.8% vs. 24.2% ± 5.9% and 50.4% ± 14.6% vs. 17.0% ± 5.6%, respectively; *p* < 0.001 in both cases; Fig. 4A), revealing partial but significant restoration of both CD4^+^ and CD8^+^ T-cell proliferation. Similar results were obtained in BM-MSC + stimulated PBMC co-cultures. Next, we examined the effect of the addition of L-NMMA, which suppresses NO production, in co-cultures of hMuStem cells + PHA-stimulated PBMCs and of BM-MSCs + PHA-stimulated PBMCs. In L-NMMA-treated co-cultures of hMuStem cells + PBMCs, the proportions of proliferative CD4^+^ and CD8^+^ T-lymphocytes were significantly higher than those observed in untreated co-cultures (73.3% ± 30.5% vs. 54.4% ± 27.2% and 72.8% ± 40.7% vs. 50.4% ± 38.8%, respectively; *p* < 0.001 in both cases; Fig. 4B). These results reflect a 21.4% ± 9.6%, and 24.3% ± 16.4% restoration of PBMC proliferation, respectively (*p* < 0.05; Fig. 4B) Importantly, the proportions of proliferating CD4^+^ and CD8^+^ T-lymphocytes were unchanged in the L-NMMA-treated BM-MSC + PBMC co-cultures, despite the fact that iNOS was expressed by BM-MSCs.

Taken together, these findings point to PGE2 as a mediator of the immunosuppressive effects of hMuStem cells on T-lymphocyte proliferation, as already described for BM-MSCs, and also indicate a role of iNOS that appears not to be shared with BM-MSCs.

### ***hMuStem cells directly inhibit CD3***^+^***T-lymphocyte proliferation in a dose-dependent manner***

To further characterize the impact of hMuStem cells on T-lymphocyte regulation, we investigated whether hMuStem cell-induced inhibition of T-cell proliferation is mediated directly by hMuStem cells, or also involves the regulation of other cells within PBMCs. CD3-sorted lymphocytes stimulated with allogeneic PBMCs in MLR were co-cultured with hMuStem cells (*n* = 4, independent donors) at T-cell:hMuStem cell ratios of 16:1 to 1:2. The addition of hMuStem cells at ratios of 16:1 and 4:1 resulted in no change in the proliferation of MLR-stimulated CD3^+^ lymphocytes relative to that observed in T-cells alone (Additional file 3: Figure S2). By contrast, the addition of hMuStem cells at ratios of 1:1 and 1:2 dramatically inhibited the proliferation of MLR-stimulated CD3^+^ lymphocytes (88.1% ± 12.6% and 99.0% ± 1.20%, respectively), demonstrating that hMuStem cells strongly suppress T-cell proliferation in a dose-dependent manner (*p* < 0.03). Importantly, these findings show that hMuStem cells can act directly on T-cells independently of the participation of other immune cell types.

### hMuStem cells inhibit the cytotoxic response of CD8^+^ T-lymphocytes through cell–cell contact and paracrine activity

**Fig. 5:**
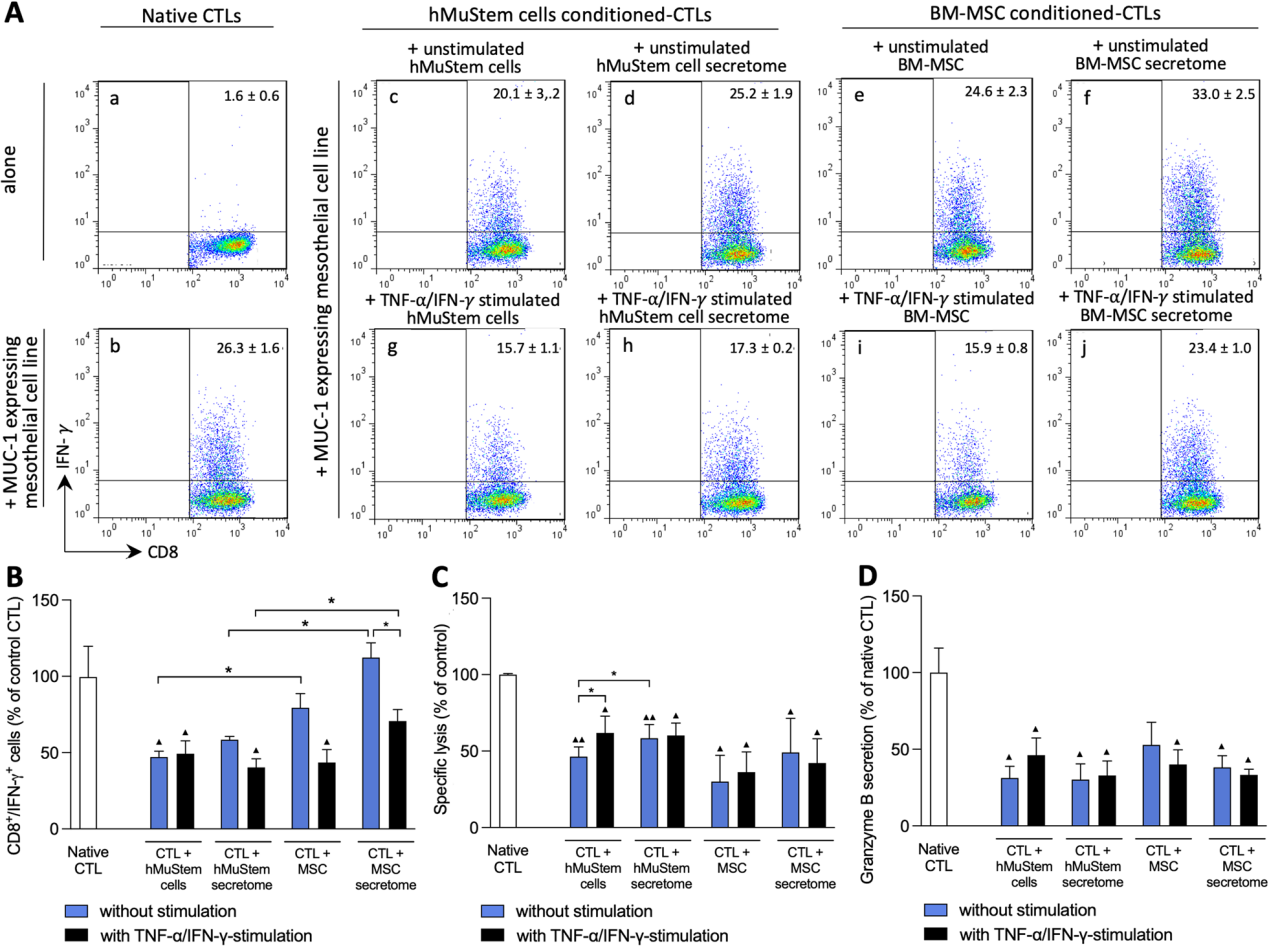
Effect of human MuStem cells and human bone-marrow mesenchymal stem cells on the cytotoxic response of CD8^+^ T-lymphocytes. **a** Flow cytometry comparison of IFN-γ expression on CD8^+^ cytotoxic T-lymphocytes (CTLs) cultured either alone or with a MUC-1-expressing mesothelioma cell line. Native CTLs correspond to CTLs cultured in standard medium. Conditioned-CTLs correspond to CTLs co-cultured with hMuStem cells or BM-MSCs (unstimulated or TNF-α/IFN-γ-stimulated) or with their corresponding secretome (collected in both conditions). Percentage of IFN-γ^+^ CD8^+^ cells from one representative experiment are reported in the right upper corner of each corresponding plot. **b** Percentage of IFN-γ^+^ cells in CTLs co-cultured with hMuStem cells or BM-MSCs (unstimulated or TNF-α/IFN-γ-stimulated) or with their corresponding secretome. **c** Quantification of specific lysis of CTLs co-cultured with hMuStem cells or BM-MSCs (unstimulated or TNF-α/IFN-γ-stimulated) or with their corresponding secretome. **d** Quantification of granzyme B secretion by CTLs cultured with a MUC-1 expressing mesothelioma cell line. CTLs were cultured in basal conditions (native CTLs) or conditioned with either hMuStem cells and BM-MSCs (unstimulated or TNF-α/IFN-γ-stimulated) or their corresponding secretome. Experiments were performed on 5 and 4 independent batches of hMuStem cells and BM-MSCs, respectively. Data are presented as mean ± SEM **(**significant difference compared to native CTLs: **▴***p* < 0.05, **▴▴***p* < 0.01; significant difference between two conditions; *p < 0.05, **p < 0.01; Mann–Whitney U test or Wilcoxon matched-pairs signed rank test (unstimulated vs. stimulated condition)

Having established that hMuStem cells can inhibit T-cell proliferation and promote a Treg cell-like phenotype, we next sought to determine their impact on the cytotoxic activity of T-lymphocytes. To this end, we examined IFN-γ expression and the cytotoxic response of a CD8^+^ cytotoxic T-lymphocyte (CTL) clone that specifically recognizes the HLA-A2/MUC1 peptide complex after contact with a luciferase-expressing mesothelioma cell line expressing this complex. First, the CTL clone was co-cultured for 6 h either directly with hMuStem cells or BM-MSCs at ratio of 1:10 (stem cells:CTLs) or with the corresponding secretome. After contact with the specific mesothelioma cell line, the proportion of IFN-γ^+^ cells was determined in the different conditions (Fig. 5A). In the co-cultures of CTLs with either hMuStem cells (panel c) or their secretome (panel d), the percentage of cells expressing IFN-γ was 47.2% ± 8.4% and 58.4% ± 4.8% of that observed with CTLs cultured alone (native CTLs; panel b), respectively (Fig. 5B). While a trend toward a reduction in the proportion of IFN-γ^+^ cells was observed in presence of hMuStem cell secretome compared to native CTLs, a significant decrease was observed only for CTLs co-cultured directly with hMuStem cells (*p* < 0.05), suggesting that suppression of CTL activation by hMuStem cells relies on a direct contact. In comparison, when BM-MSCs (panel e) or their secretome (panel f) were added to CTLs, the proportion of cells expressing IFN-γ corresponded to 79.4% ± 18.7% and 112.4% ± 19.1% of that observed in native condition, revealing an absence of any inhibitory action from BM-MSC on CTL activation. Given that hMuStem cells and BM-MSCs enhance the expression and secretion of regulatory molecules in pro-inflammatory conditions, we next repeated the cytotoxicity assay using TNF-α/IFN-γ-stimulated cells. Compared to CTLs co-cultured with unstimulated hMuStem cells, no changes were observed in CTLs cultured in presence of stimulated hMuStem cells (panel g). However, a significant decrease of IFN-γ^+^ cells was noted with stimulated hMuStem cell secretome (representing decreases of 2.0-fold and 2.4-fold, respectively; *p* < 0.05; panel h). In presence of stimulated BM-MSCs (panel i), the percentage of IFN-γ^+^ cells was significantly reduced (43.6% ± 16.9% of that observed in CTLs cultured alone) showing the ability of BM-MSCs to potently suppress the activation of CTL through direct contact (*p* < 0.05). The percentage of IFN-γ^+^ cells was lower when CTLs were co-cultured with stimulated BM-MSC secretome as compared to the unstimulated BM-MSC secretome (70.7% ± 15.2% vs 112.4% ± 19.1% of native CTLs; *p* < 0.05; panel j). Altogether, those results show that, in contrast to hMuStem cells, BM-MSCs require a TNF-α/IFN-γ stimulation to suppress CTL activation through both direct cell contact and the secretion of soluble factors.

Luciferase activity in supernatant of co-cultures revealed specific lysis in CTLs co-cultured with hMuStem cells and those co-cultured with hMuStem cell secretome, corresponding to 46.4% ± 16.7% and 58.4% ± 23.9% with respect to the native condition (Fig. 5C). These results reveal lower cytotoxic activity in conditioned CTLs than native CTLs (*p* < 0.01 and *p* < 0.05, respectively), although this reduction was significantly more pronounced when CTLs were in direct contact with hMuStem cells (*p* < 0.05). In CTLs co-cultured with BM-MSCs or their secretome, lytic activity corresponded to 30.1% ± 34.3% and 49.1% ± 44.6% of native condition, respectively, revealing a lower intensity in both conditions (*p* < 0.05). Thus, inhibition of CTL cytotoxic activity induced by hMuStem cells and BM-MSCs appears to be mediated by cell–cell contact-dependent mechanisms as well as secreted factors. Interestingly, no statistical differences in the intensity of inhibition of CTL-mediated lysis were observed either between unstimulated and TNF-α/IFN-γ stimulated conditions or between hMuStem cells and BM-MSCs. In line with this view, secretion of granzyme B by CTLs was markedly reduced when they were co-cultured with either hMuStem cells or their secretome (Fig. 5D). Similar results were observed in co-cultures performed with BM-MSCs and their secretome. Also, TNF-α/IFN-γ stimulation did not alter the degree of inhibition of granzyme B secretion in both cultures done with hMuStem cells and BM-MSCs. Overall, these findings demonstrate that naïve hMuStem cells, unlike BM-MSCs that require a pro-inflammatory stimulation, reduce the cytotoxic response of CTLs, through mechanisms that involve both cell–cell contact and soluble factors.

## Discussion

In this study, we provide original and compelling data demonstrating the capacity of hMuStem cells to control T-cell function, highlighting the potent immunosuppressive properties of these cells. Our data indicate that hMuStem cells exhibit a poorly immunogenic phenotype and also interact with T-cells to regulate their functions. We show that hMuStem cells can (1) induce the generation of Treg cells; (2) inhibit CD4^+^ and CD8^+^ T-lymphocyte proliferation in part by regulating PGE2 secretion and iNOS activity; and (3) repress the CTL response both through direct cell–cell contact and paracrine factors.

The present study provides the first evidence that hMuStem cells and BM-MSCs share a similar immunophenotype. Indeed, in basal conditions, hMuStem cells are positive for HLA-I molecules and are negative for HLA-II (*i.e.,* HLA-DQ, HLA-DR) and for co-stimulatory molecules, including CD80, CD86 and CD40, that are required for complete T-cell activation. We found that 10–60% of hMuStem cells were HLA-DP^+^. Our data suggest that hMuStem cells, like BM-MSCs, have a poorly immunogenic profile. Moreover, we found that stimulation of hMuStem cells with TNF-α/IFN-γ upregulated HLA-I molecules and uniformed HLA-DP expression. These observations distinguish hMuStem cells from BM-MSCs, in which HLA-DR is upregulated in response to pro-inflammatory stimulation [74]. The phenotype described here for hMuStem cells is also consistent with the characterization of Mabs, for which immunomodulatory properties have been described [78]. Mabs constitutively express HLA-ABC and low levels of HLA-DR in the resting state, and express neither the co-stimulatory molecules CD40, CD80 or CD86 nor the inhibitory molecules PDL-1 or cytotoxic T-lymphocyte antigen-4. They also observed increased expression of HLA-DR, but not HLA-ABC after IFN-γ stimulation. BM-MSCs exert their immunosuppressive function through the expression of a wide number of membrane molecules, including PDL-1 and PDL-2, which are ligands of PD-1 expressed by T-lymphocytes [76, 79, 80]. PD-1 signal transduction pathways in T-cells mediate the inhibition of proliferation, hyporesponsiveness and apoptosis [81]. We found that hMuStem cells did not express PDL-1, but were homogeneously positive for PDL-2, which negatively regulates T-cell responses and plays a pivotal role in immune tolerance [82]. Moreover, stimulation with TNF-α/IFN-γ induced the expression of PDL-1 while up-regulating PDL-2, indicating that hMuStem cells have a favorable phenotypic profile for the modulation of T-cell activity.

Human MuStem cells express the main lymphocyte adhesion molecules ICAM-1 and VCAM-1, which are crucial for leukocyte adhesion and for the formation and stabilization of immunological synapses between T-cells and APCs [83]. ICAM-1 and VCAM-1 are also critical for MSC-mediated immunosuppression [84]. ICAM-1 signaling increases the immunosuppressive capacity of MSCs by enhancing PGE2 secretion and IDO expression [85]. Moreover, VCAM-1-positive MSCs exert a greater suppressive effect than VCAM-1-negative MSCs by inhibiting the Th1 response and inducing the generation of Treg cells [86]. We found that in basal conditions hMuStem cells, like BM-MSCs, expressed low levels of ICAM-1 and VCAM-1, both of which increased after exposure to pro-inflammatory cytokines (TNF-α/IFN-γ), as evidenced by detection of 100% of positive cells and a 27-fold increase in ICAM-1 expression. This expression profile could explain the immunoregulatory properties of hMuStem cells, their capacity to physically interact with T-cells and thereby reinforce inhibitory molecule-mediated signaling (i.e., via PDL-1, PDL-2, CD155), and the proximity of T-cells to secreted immunosuppressive mediators.

Human MuStem cells inhibited T-cell proliferation when co-cultured with a mix of PBMCs or isolated activated T cells, revealing a direct inhibitory effect of hMuStem cells (i.e., without the involvement of other immune cell partners). These observations are consistent with the findings of multiple studies showing that human MSCs directly suppress T-cell proliferation in a dose-dependent manner [39, 87]. Human Mabs have also been shown to potently and dose-dependently suppress CD4^+^ and CD8^+^ T-cell proliferation in vitro, exerting a significant inhibitory effect at Mabs:PBMC ratios of between 1:1 and 1:4 [88]. Our investigation of the underlying mechanisms revealed the involvement of PGE2, as previously reported for both MSCs [89] and Mabs [78]. We also uncovered a role of iNOS activity in the inhibitory activity of hMuStem cells, but not BM-MSCs, highlighting a major difference between these two cell types. We found that BM-MSCs were uniformly positive for iNOS expression, in contrast to the findings of several studies demonstrating that iNOS is not expressed by human MSCs but is expressed in MSCs derived from other species including mouse, rat, hamster and rabbit [90].

Another key finding of our study is the capacity of hMuStem cells to specifically interact with CTLs and suppress their cytotoxic response. Indeed, after 24 h of contact with hMuStem cells, CTL clones showed a decrease in their ability to express IFN-γ, secrete granzyme B and lyse target cells following recognition of a specific HLA-I/peptide signal. These findings indicate that hMuStem cells inhibit the activation and function of fully differentiated IL-2-activated clonally-derived CTLs. Importantly, this global inhibitory effect is constitutive for hMuStem cells whereas it is for BM-MSCs. In line with this finding, an inhibitory effect was observed when BM-MSCs were co-cultured with PBMCs, but only when they were added prior to CTL activation or during the early phase of CTL activation and in a dose-dependent manner [91]. This finding could suggest that the lack of effect observed here on IFN-γ expression could be related to a lesser action of BM-MSCs compared to hMuStem cells. In addition, it would be interesting to further explore this ability of hMuStem cells by studying their effect on the activity of NK cells, which are constitutively cytotoxic. This would be all the more interesting as studies of the inhibitory effects of BM-MSCs on NK cells have produced conflicting findings, ranging from no effects [91] to reduced cytotoxic activity of NK cells [44].

The co-culture of PBMCs or CTLs with hMuStem cell secretome instead of hMuStem cells produced similar, albeit milder, effects, suggesting that the inhibitory effect of hMuStem cells involves paracrine activity but also requires direct cell–cell contact. A role of direct contact is supported by the constitutive and high-level expression of CD155 and ICAM-1, respectively, observed in all hMuStem cells. Pro-inflammatory stimulation is known to increase both the secretion of immunomodulatory mediators and the expression of the majority of molecules involved in interactions with lymphocytes. Notably, CTLs previously cultured with stimulated hMuStem cells showed lytic capacities and IFN-γ expression levels similar to those of CTLs cultured with naïve hMuStem cells. This may be explained by the fact that the co-culture environment already provides an inflammatory signal to the hMuStem cells due to the presence of IFN-γ produced by CTLs. An alternative hypothesis is that the membrane molecules that induce CTL inhibition are not modulated by IFN-γ or TNF-α, as described for CD155. For further comparison between hMuStem cells and BM-MSCs, it would be informative to determine why TNF-α/IFN-γ stimulation is instead required for BM-MSCs to reduce IFN-γ expression by CTLs. The secretome of stimulated hMuStem cells did not further decrease granzyme B secretion or the lytic capacity of CTLs relative to naïve hMuStem cells but did result in lower IFN-γ expression in CTLs after activation by the HLA-I/peptide complex. This indicates that prior stimulation of hMuStem cells leads to a greater paracrine effect on IFN-γ expression but not on specific lytic activity of CTLs. Of note, a similar impact with a more marked intensity was observed for the secretome of stimulated BM-MSCs. This result suggests that the hMuStem cells use distinct paracrine mechanisms of action to inhibit IFN-γ expression and to suppress the lytic capacity of CTLs. Consistent with this view, IFN-γ production appears not to be directly correlated with cytotoxic function [92]. Together, these findings highlight the diversity of modes of action used by hMuStem cells to suppress the cytotoxic response of CTLs, a key characteristic that appears to distinguish hMuStem cells from BM-MSCs.

Here, we found that such as BM-MSCs [51, 60], hMuStem cells induced the generation of Treg-like cells corresponding to Tr-1 and CD4^+^FoxP3^+^ cells. However, in our experimental conditions, the capacity of hMuStem cells to induce Treg cells was significant but modest, resulting in the generation of few Tr1 and CD4^+^FoxP3^+^ cells. Therefore, it will be of interest to further elucidate the immunoregulatory mechanisms of hMuStem cells by studying the regulatory function of Tr1 and CD4^+^FoxP3^+^ cells generated at the end of co-culture experiments. This type of functional study is warranted given that Treg cells play a critical role in the pathogenesis of MDs, as evidenced by the direct relationship between the increase in Treg cell number and the reduction in both inflammation and fiber necrosis in dystrophic mice [17, 18].

Finally, our findings provide evidence that LIF secretion clearly distinguishes hMuStem cells from BM-MSCs. LIF has been proposed to play a key role in the generation of Treg cells [93, 94]. Indeed, specific blockade of the glycoprotein cytokine LIF in co-cultures of human BM-, Wharton’s jelly- and adipose tissue-derived MSCs + PBMCs resulted in restoration of CD3^+^ lymphocyte proliferation by up to 91% and a decrease in CD4^+^CD25^+^Foxp3^+^ cells. Given the inhibitory effect of hMuStem cells on T-cell proliferation and their capacity to generate Tregs described here, it would be informative to further examine the role of LIF in the immunosuppressive effect of hMuStem cells.

## Conclusion

Our findings show that hMuStem cells exert potent immunosuppressive properties on T-cells, inhibiting their proliferation and cytotoxic response and inducing the generation of Tregs. Human MuStem cells may modulate immune cell activity in addition to directly contribute to muscle fiber formation/regeneration, thereby exerting positive effects on the pathophysiology of MDs through two complementary modes of action. Human MuStem cells could therefore constitute a particularly efficient therapeutic tool for clinical application in the context of MDs.

## Supplementary Information

**Additional file 1: Methods S1.** Immunosuppression assay.

**Additional file 2: Table S1.** List of antibodies used for flow cytometry analysis.

**Additional file 3: Table S2.** Primers used for RT-qPCR analysis.

**Additional file 4: Figure S1.** Expression profile for cell lineage-specific surface markers by human MuStem cell.

**Additional file 5: Figure S2.** Dose effect of human MuStem cells on the proliferation of CD3+ lymphocytes cultured with irradiated peripheral blood mononucleated cells.

## Data Availability

The datasets used and/or analyzed during the current study to support the conclusions are included in the article and in the corresponding additional files. They can be made available from the corresponding author on reasonable request.

## Abbreviations

7-AAD: 7-Amino-actinomycin D
α-MEM: Alpha-minimum essential medium
Ab: Antibody
APC: Antigen-presenting cell
AD-MSC: Adipose tissue-derived mesenchymal stem cell
bFGF: Basic fibroblast growth factor
BM-MSC: Bone marrow-derived mesenchymal stem cell
CTV: Cell trace violet
DC: Dendritic cell
DMD: Duchenne muscular dystrophy
EGF: Epidermal growth factor
FCS: Fetal calf serum
Gal-1: Galectin-1
GM: Growth medium
GRMD: Golden retriever muscular dystrophy
HGF: Hepatocyte growth factor
HLA: Human leukocyte antigen
HO-1: Heme oxygenase-1
IDO: Indoleamine 2,3-dioxygenase
IFN: Interferon
IMDM: Iscove’s modified Dulbecco’s medium
iNOS: Inducible nitrite oxide synthase
IL: Interleukin
LIF: Leukemia inhibitory factor
L-NMMA: NG-monomethyl-L-arginine
Mabs: Mesoangioblasts
MD: Muscular dystrophy
MDSC: Muscle-derived stem cell
*mdx*: X-chromosome-linked muscular dystrophy
MLR: Mixed lymphocyte reaction
mRNA: Messenger ribonucleic acid
MSC: Mesenchymal stem cell
NEAA: Non-essential amino-acid
NK: Natural killer
NO: Nitric oxide
PBMC: Peripheral blood mononuclear cell
PBS: Phosphate buffered saline
PGE2: Prostaglandin E2
PHA: Phyto-hemagglutinin
PICs: PW1+ Pax7+ interstitial cells
PMA: Phorbol myristate acetate
PSF: Penicillin–streptomycin-fungizone
RT-PCR: Reverse transcription-polymerase chain reaction
SCID: Severe combined-immunodeficiency
SP: Side population
TNF: Tumor necrosis factor
Treg: Regulatory T
VEGF: Vascular endothelial growth factor
