# Supplementary material for: Human MuStem cells repress T-cell proliferation and cytotoxicity through both paracrine and contact-dependent pathways
Source: Stem Cell Res Ther. 2022 Jan 10;13:7. doi: 10.1186/s13287-021-02681-3 (PMC8751303; doi:10.1186/s13287-021-02681-3)
Supplement: Supplementary file 1 — Additional file 1: Methods S1. Immunosuppression assay. [file 13287_2021_2681_MOESM1_ESM.docx]

SUPPLEMENTAL FIGURE LEGENDS

**Figure S1.** Expression profile for cell lineage-specific surface markers by human MuStem cell. Flow cytometry profiles of one representative cell batch out of 5 independent cell batch presented. Expression of (A) myogenic progenitor markers; (B) endothelial and perivascular cell lineage markers; (C) canonical mesenchymal stem cell (MSC) markers; (D) hematopoietic stem cell (HSC) and immune cell markers, were evaluated. Cultured hMuStem cells expressed the canonical satellite cell (SC) marker CD56 (28% to 69%) whereas all cells were robustly positive for the recently identified SC marker CD29. Human MuStem cells were uniformly negative for the expression of the markers CD31 and CD144 typically expressed by endothelial lineage cells. The well-known perivascular cell markers CD140b and CD146 were expressed by 60.7% ± 28.2% and 43.6% ± 30.9% of the hMuStem cells, respectively. As expected, the canonical MSC markers CD44, CD73, CD90 and CD166 were homogenously observed in hMuStem cells that were concomitantly negative for the classical HSC markers CD34 and CD45 as well as the blood lineage markers CD14 and CD19. Isotype controls and specific signals are shown in white and gray, respectively.

**Figure S2.** Dose effect of human MuStem cells on the proliferation of CD3^+^ lymphocytes cultured with irradiated peripheral blood mononucleated cells. CD3^+^ lymphocytes were co-cultured with irradiated allogeneic peripheral blood mononucleated cells (PBMCs) at a 1:1 ratio for 5 days in the absence or presence of increasing numbers of irradiated hMuStem cells. Proliferation was assessed based on the incorporation of tritiated thymidine. Results were obtained with hMuStem cells from 4 different batches in independent experiments. Data are presented as the mean ± SEM (**p* <0.03).
