# Supplementary figures and images for: Human MuStem cells repress T-cell proliferation and cytotoxicity through both paracrine and contact-dependent pathways
Source: Stem Cell Res Ther. 2022 Jan 10;13:7. doi: 10.1186/s13287-021-02681-3 (PMC8751303; doi:10.1186/s13287-021-02681-3)

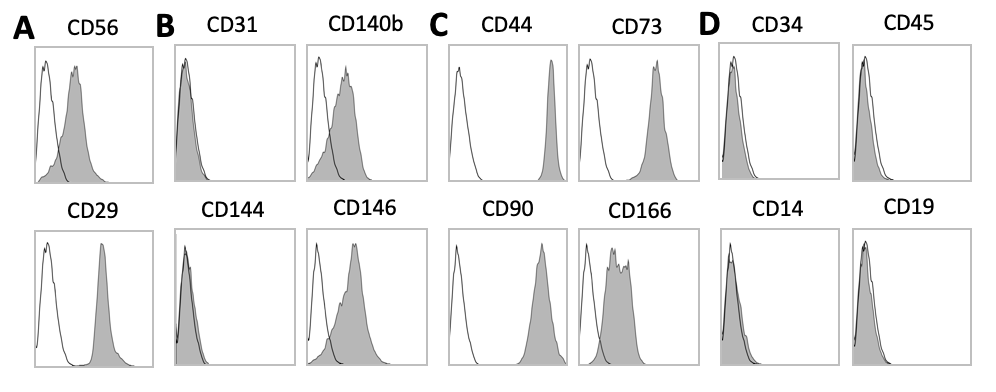

Supplement: Supplementary file 2 — Additional file 2: Table S1. List of antibodies used for flow cytometry analysis. [file 13287_2021_2681_MOESM2_ESM.tiff]

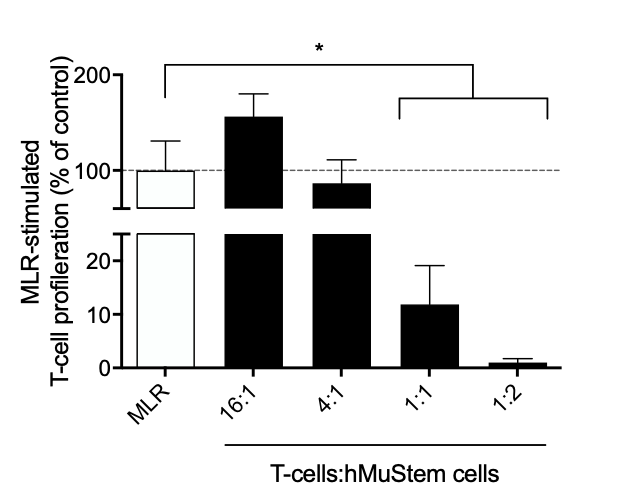

Supplement: Supplementary file 3 — Additional file 3: Table S2. Primers used for RT-qPCR analysis. [file 13287_2021_2681_MOESM3_ESM.tiff]
