# Supplementary material for: Human MuStem cells repress T-cell proliferation and cytotoxicity through both paracrine and contact-dependent pathways
Source: Stem Cell Res Ther. 2022 Jan 10;13:7. doi: 10.1186/s13287-021-02681-3 (PMC8751303; doi:10.1186/s13287-021-02681-3)
Supplement: Supplementary file 4 — Additional file 4: Figure S1. Expression profile for cell lineage-specific surface markers by human MuStem cell. [file 13287_2021_2681_MOESM4_ESM.docx]

**SUPPLEMENTAL TABLES**

**Table S1** List of antibodies used for flow cytometry analysis.

AF, alexafluor; APC, allophycocyanine; Cy, cyanine; FITC, fluorescein isothiocynate; PE, phycoerythrin; PerCP, peridinin chlorophyll protein complex, NA, not applicable.

| Antigen | Conjugation | Company and reference | Corresponding isotype |
| --- | --- | --- | --- |
| *Stem cells* | |  |  |
| CD40 | PE | BD Bioscience, 560963 | Mouse IgG1-PE |
| CD54/ICAM-1 | PE | BD Bioscience, 555511 | Mouse IgG1-PE |
| CD80 | PE | BD Bioscience, 557227 | Mouse IgG1-PE |
| CD86 | PE | BD Bioscience, 555658 | Mouse IgG1-PE |
| CD106/ VCAM-1 | PE | BD Bioscience, 555647 | Mouse IgG1-PE |
| CD112 | PE | BD Bioscience, 551057 | Mouse IgG1-PE |
| CD155 | AF647 | BD Bioscience, 556305 | Mouse IgG1-PE |
| CD273/PDL-2 | APC | BD Bioscience, 557926 | Mouse IgG1-APC |
| CD274/PDL-1 | FITC | BD Bioscience, 558065 | Mouse IgG1-FITC |
| HLA-ABC | PE | eBioscience, 129983 | Mouse IgG2a-PE |
| HLA-DP | Purified | Serotec, MCA5677 | Mouse IgG1 |
| HLA-DQ | FITC | BD Bioscience, 555563 | Mouse IgG2a-FITC |
| HLA-DR | PE | eBioscience, 129956 | Mouse IgG2b-PE |
| HLA-E | PE | BioLegend, 342603 | Mouse IgG1-PE |
| HLA-G1 | PE | BioLegend, 335905 | Mouse IgG2a-PE |
| HO-1 | AF647 | BD Bioscience, 566391 | Mouse IgG1-AF647 |
| Mouse IgG1 | AF647 | BD Bioscience, 557714 | NA |
| Mouse IgG1 | APC | BD Bioscience, 555751 | NA |
| Mouse IgG1 | FITC | BD Bioscience, 556649 | NA |
| Mouse IgG1 | PE | BD Bioscience, 555749 | NA |
| Mouse IgG1 | PE-Cy7 | BD Bioscience, 557872 | NA |
| Mouse IgG1 | PerCP-Cy5.5 | BD Bioscience, 550795 | NA |
| Mouse IgG2a | FITC | BD Bioscience, 555573 | NA |
| Mouse IgG2a | PE | R&D System, IC003P | NA |
| Mouse IgG2b | PE | BD Bioscience, 555743 | NA |
| *PBMCs* | |  |  |
| CD4 | APC | BD Pharmingen, 561840 | Mouse IgG1-APC |
| CD8 | PE | BD Pharmingen, 561949 | Mouse IgG1-PE |
| CD25 | PE-Cy7 | BD Pharmingen, 335789 | Mouse IgG1-PE-Cy7 |
| FoxP3 | PE | BD Pharmingen, 560852 | Mouse IgG1-PE |
| IFN-𝛾 | FITC | BD Pharmingen, 561057 | Mouse IgG1-FITC |
| IL-17A | PerCP-Cy5.5 | BD Pharmingen, 560799 | Mouse IgG1-PerCP-Cy5.5 |
| *CTL* | | |  |
| CD8 | APC | BioLegend, 344722 | Mouse IgG1-APC |
| IFN-𝛾 | PE | BD Bioscience, 557074 | Mouse IgG1-PE |

**Table S2** Primers used for RT-qPCR analysis.

| Gene symbol |  | Sequence | Product (bp) | Accession number |
| --- | --- | --- | --- | --- |
| *RPS18* | For | 5'-ACCAAGAGGGCGGGAGAA-3' | 85 | NM_022551.2 |
|  | Rev | 5'-CTGGGATCTTGTACTGGCGTG-3' |  |  |
| *IDO-1* | For | 5'- AAAGGCAACCCCCAGCTATC -3' | 315 | NM_002164.5 |
|  | Rev | 5'- CAGGGAGACCAGAGCTTTCACA -3' |  |  |
