# Supplementary material for: Human MuStem cells repress T-cell proliferation and cytotoxicity through both paracrine and contact-dependent pathways
Source: Stem Cell Res Ther. 2022 Jan 10;13:7. doi: 10.1186/s13287-021-02681-3 (PMC8751303; doi:10.1186/s13287-021-02681-3)
Supplement: Supplementary file 5 — Additional file 5: Figure S2. Dose effect of human MuStem cells on the proliferation of CD3+ lymphocytes cultured with irradiated peripheral blood mononucleated cells. [file 13287_2021_2681_MOESM5_ESM.docx]

**SUPPLEMENTAL METHODS**

**Immunosuppression assay**

Allogeneic human PBMCs were isolated from 3 different donors and labeled with 5 µM Cell Trace Violet (CTV; Life-Technology, Carlsbad, CA, USA) by incubation for 20 min at 37°C. PBMCs were centrifuged and resuspended in Iscove's Modified Dulbecco's Medium (IMDM; Gibco) supplemented with 10% fetal calf serum (FCS, Pierce-Hyclone, Logan, UT, USA), 2 mM L-glutamine (Lonza), 1% 10,000 IU/mL penicillin, 10 mg/mL streptomycin, 25 µg/mL fungizone (amphotericin B) (PSF; Sigma-Aldrich, Saint Quentin-Fallavier, France), 100 µM non-essential amino-acid (NEAA) (Lonza), 1 mM pyruvate (Gibco), 20 mM Hepes (Gibco), and 50 µM β-mercaptoethanol (Gibco). PBMCs (2 × 10^5^) were stimulated with 5 µg/mL phytohemagglutinin (PHA, R&D Systems) and co-cultured with 2 × 10^4^ unstimulated hMuStem cells or BM-MSCs (1:10 ratio) in a 96-well plate. For PGE2 and iNOS inhibition experiments, either 0.1 mM indomethacin (Sigma-Aldrich) or 1 mM NG-monomethyl-L-arginine (L-NMMA; Sigma-Aldrich) was added to the co-cultures. PBMCs were stimulated with phorbol myristate acetate (PMA) (50 ng/mL; Merck, Darmstadt, Germany) and ionomycin (1 mg/mL; Merck) in the presence of brefeldin A (10 mg/mL; Merck) for 5 hours. Lymphocyte proliferation and phenotype were analyzed by flow cytometry after 3 days. Cells were stained with the LIVE/DEAD Fixable near-IR stain (Invitrogen, Cergy-Pontoise, France) to analyze live cells only. To examine proliferation behavior, cells were incubated with antibodies (Ab) against CD4 and CD8 (Table S1) and specific CD4^+^ and CD8^+^ T-lymphocyte proliferation was analyzed by measuring CTV intensity. For differentiation analysis, cells were incubated with Ab against CD25 and then fixed, permeabilized at 4°C with Cytofix/Cytoperm buffer (BD Pharmingen, Franklin Lakes, NJ, USA), and labeled with intracellular fluorochrome-conjugated Ab against IFN-γ, interleukin (IL)-10, IL-17, and FoxP3 (Table S1) diluted in Perm/Wash buffer (eBioscience, Montrouge, France) according to the manufacturer’s specifications.

In a second set of experiments, MLR analyses were performed. Human CD3^+^ lymphocytes were isolated by centrifugal counter-flow elutriation from blood samples of patients (Clinical Transfer Facility, CICBT0503, Nantes, France) and sorted by negative magnetic sorting using EasySepTM kit (>90% purity; Stemcell Technologies, Vancouver, Canada). CD3^+^ lymphocytes were seeded in 96-well plates (10^5^ cells/well) with irradiated allogeneic PBMCs (10 min, 35 Gy) at a 1:1 ratio. Irradiated hMuStem cells (10 min, 35 Gy) were added to achieve T cell:hMuStem cell ratios of 16:1; 4:1; 1:1, and 1:2. After 5 days, the cultures were incubated overnight with tritiated thymidine (0.925 µBq/mL, Perkin Elmer, Zaventem, Belgium) and cells were harvested on filter using a Harvester Mach III (Tomtec, Hamden, USA). Proliferation was detected by measuring radioactivity using the 1450 MicroBeta Jet (Perkin Elmer). The activation threshold was determined by considering the mean proliferation of unstimulated lymphocytes. The percentage proliferation was calculated as follows: Percentage inhibition = 100 – (radioactivity measured in sample / radioactivity measured in activated T-cell control) *100.

To analyze the effect of hMuStem cells or BM-MSCs on CTLs, HLA-A2*0201/MUC1(950-958)-specific CD8^+^ T-cell clones, previously obtained by limiting dilution [95], were expanded in RPMI 1640 (Gibco) containing 8% human serum and 150 IU/mL IL-2, and stimulated with irradiated (35 Gy) feeder cells. Human MuStem cells or BM-MSCs expanded in basal or TNF-α/IFN-γ-stimulated conditions were harvested and plated in a 96-well plate for 24 hours. For direct contact experiments, clonally-derived CD8^+^ T-cells were added and co-cultured for 6 hours at a hMuStem cell or BM-MSCs:T-cell ratio of 1:4. For indirect contact experiments, the conditioned medium from hMuStem cells or BM-MSCs (corresponding to the supernatant from a culture of 2 × 10^4^ cells expanded for 24 hours) was recovered and added to 8 × 10^4^ clonally-derived T-cells for 6 hours. Clonally-derived T-cells were then harvested and 5 × 10^4^ cells were incubated for 5 hours at 37°C with 10^5^ HLA-A2 Meso 34 cells [96] in complete medium containing 10 mg/mL brefeldin A (Sigma-Aldrich) and subsequently washed. Cells were labeled with allophycocyanin-conjugated mouse anti-human CD8 (BD Biosciences, Le Pont de Claix, France). After 2 washes with PBS, cells were then fixed with PBS containing 4% paraformaldehyde for 10 min at room temperature. Cell membranes were permeabilized with PBS containing 0.1% BSA (Sigma-Aldrich) and 0.1% saponin (Sigma-Aldrich) for 5 min, washed twice with PBS, and incubated for 30 min at room temperature with PE-conjugated mouse anti-human IFN-γ mAb (BD Biosciences). After 2 washes with PBS, IFN-γ expression was determined by flow cytometry gated for CD8^+^ T-cells.
